# Interventions to Mitigate Emergency Department and Hospital Crowding During an Infectious Respiratory Disease Outbreak: Results from an Expert Panel

**DOI:** 10.1371/currents.dis.1f277e0d2bf80f4b2bb1dd5f63a13993

**Published:** 2013-04-17

**Authors:** Andrea Freyer Dugas, Melinda Morton, Raphaelle Beard, Jesse M. Pines, Jamil D. Bayram, Yu-Hsiang Hsieh, Gabor Kelen, Lori Uscher-Pines, Kevin Jeng, Gai Cole, Richard Rothman

**Affiliations:** Department of Emergency Medicine, Johns Hopkins University, Baltimore, Maryland, United States; Department of Emergency Medicine, Johns Hopkins University, Baltimore, Maryland, United States; Department of Emergency Medicine, Johns Hopkins University, Baltimore, Maryland, United States; Departments of Emergency Medicine and Health Policy, George Washington University, Washington, District of Columbia, United States; Department of Emergency Medicine, Johns Hopkins School of Medicine, Baltimore, Maryland, USA; Department of Emergency Medicine, Johns Hopkins University, Baltimore, Maryland, United States; Department of Emergency Medicine, Johns Hopkins University, Baltimore, Maryland, United States; RAND Corporation, Arlington, Virginia, United States; Department of Emergency Medicine, Johns Hopkins University, Baltimore, Maryland, United States; Department of Emergency Medicine, Johns Hopkins University, Baltimore, Maryland, United States; Department of Emergency Medicine, Johns Hopkins University, Baltimore, Maryland, United States

## Abstract

Objective: To identify and prioritize potential Emergency Department (ED) and hospital-based interventions which could mitigate the impact of crowding during patient surge from a widespread infectious respiratory disease outbreak and determine potential data sources that may be useful for triggering decisions to implement these high priority interventions. Design: Expert panel utilizing Nominal Group Technique to identify and prioritize interventions, and in addition, determine appropriate “triggers” for implementation of the high priority interventions in the context of four different infectious respiratory disease scenarios that vary by patient volumes (high versus low) and illness severity (high versus low). Setting: One day in-person conference held November, 2011. Participants: Regional and national experts representing the fields of public health, disease surveillance, clinical medicine, ED operations, and hospital operations. Main Outcome Measure: Prioritized list of potential interventions to reduce ED and hospital crowding, respectively. In addition, we created a prioritized list of potential data sources which could be useful to trigger interventions. Results: High priority interventions to mitigate ED surge included standardizing admission and discharge criteria and instituting infection control measures. To mitigate hospital crowding, panelists prioritized mandatory vaccination and an algorithm for antiviral use. Data sources identified for triggering implementation of these interventions were most commonly ED and hospital utilization metrics. Conclusions: We developed a prioritized list of potentially useful interventions to mitigate ED and hospital crowding in various outbreak scenarios. The data sources identified to “trigger” the implementation of these high priority interventions consist mainly of sources available at the local, institutional level.

## Introduction

As the safety net of the U.S. healthcare system, emergency departments (ED) are responsible for managing the large influx of patients, commonly referred to as a “surge”, resulting from a disaster or pandemic outbreak. However, EDs are already stretched to near-capacity and are routinely overcrowded.^1^ ED crowding itself, apart from disasters, is associated with decreased quality of care, including delays in critical treatments and increased risks of in-hospital mortality.^2^^,^ 3 ^,^ 4 ^,^ 5 This crowding is exacerbated by seasonal influenza, and many hospitals and EDs are unprepared to manage the surge in patient volumes which occur during a large scale infectious respiratory disease outbreak.^1^ Even during the 2009 H1N1 pandemic influenza outbreak, when the majority of patients were not critically ill and did not require intensive ED or hospital services, many EDs and hospitals across the U.S. were overwhelmed and unable to meet patient care demands in a timely manner.^6^^,^ 7 ^,^ 8 ^,^ 9 ^,^ 10

To maintain high-quality care and manage the increase in ED volumes during an infectious respiratory disease outbreak, hospitals and EDs must have a response plan with the ability to implement timely and effective interventions designed to increase capacity and diminish the effects of crowding which can directly impact patient safety and quality of care. Although there are numerous reports in the literature describing various interventions designed to mitigate ED and hospital crowding due to infectious respiratory disease outbreaks, it is difficult to compare the impact of interventions across sites due to lack of standardized outcome measures, as well as variability in medical center structure, patient populations, and the particular characteristics and dynamics of an individual outbreak.^11^^,^ 12 ^,^ 13 ^,^ 14 Furthermore, medical centers tend to implement multiple responses during an outbreak to help control both short and long-term patient surges, further complicating attempts to independently and accurately evaluate the impact of any individual intervention on the outcomes of interest, namely maintaining timely care within the ED and hospital.

While many hospitals across the U.S. have preparedness plans and protocols for dealing with emerging infections, not all outbreaks are the same. For example, outbreaks can manifest as high or low severity of illness, and high or low levels of contagion, which ultimately contributes to the volume of patients seeking care. Hence, it is unclear if a single set of preparedness protocols would address varying types of outbreaks with differential volume of patients and severity of illness. Given the inherent difficulty of analyzing the published literature, and lack of differentiation in the operational approach to the various types of major emerging infectious outbreaks, it is necessary to approach this problem through a mixed methods approach.

Utilizing the opinions of an expert panel, we compared and prioritized ED and hospital interventions to mitigate the impact of a surge due to infectious respiratory disease outbreaks with varying patient volume and severity of illness. A secondary objective was to prioritize potential data sources which might trigger the decision to implement ED or hospital-based interventions for each of the outbreak types.

## Methods

**Expert Panel Selection**

We convened an expert panel for a one-day conference on November 4th, 2011 in Baltimore, Maryland. Panelists with direct experience and expertise in fields which contribute to the guidance, decision-making, and implementation of local and national interventions associated with mitigation of ED and/or hospital surge were selected by the advisory panel based upon their expertise in the field as evidenced by their record of publication, speaking engagements at national meetings, impact on national or regional policy, or representation of a directly related governmental organization. Of the 66 panelists invited, 34 (52%) attended the one day conference and were offered financial reimbursed for basic travel expenses only. To ensure appropriate representation, participants were selected from all key geographic areas of the U.S., and if a representative of a directly related governmental organization was unable to attend, they were asked to select a replacement to represent that organization. The panelists represented the fields of public health (8), disease surveillance (15), clinical medicine (13), emergency medicine operations (8), hospital operations (6), and systems experts (7), with some panelists representing more than one field. Representatives included those from the federal organizations (2), state and city health departments (6), corporate and non-profit organizations (2), and academic institutions (24). This study was reviewed and approved by the Institutional Review Board with a waiver of consent.

**Initial Survey**

To accomplish our objectives, we used a mixed-methods design, involving an initial survey instrument followed by in-person discussion utilizing nominal group technique.^15^^,^ 16 Prior to the conference, the study team members (AD, MM, RB, KJ, RR) performed a review of the existing academic literature to identify potential ED and hospital-based interventions to manage crowding from a respiratory infectious disease outbreak.

This initial list of potential interventions was distributed to the 34 invited panelists who committed to attend the conference one month in advance. In addition, study investigators distributed definitions and brief descriptions of four types of infectious respiratory disease outbreaks to allow the panelists the opportunity to express their varied opinions on interventions relative to expected patient volume and severity of illness. These four scenarios were: 1) low volume/low severity (e.g. seasonal influenza), 2) high volume/low severity (e.g. 2009 H1N1), 3) low volume/high severity (e.g. Severe Acute Respiratory Syndrome [SARS]), and 4) high volume/high severity (e.g. 1918 H1N1).

Panelists were instructed to separately rate each intervention by both ease of implementation and importance for each of the four infectious respiratory disease outbreak scenarios using a 1-5 Likert scale. Ease of implementation included: consideration of factors such as cost, operational complexity, facility (time) of setup, and intensity of resource utilization. Importance was defined as the likely effectiveness of the intervention in reducing ED or hospital crowding or augmenting surge. Panelists were encouraged to suggest additional “write-in” interventions not already listed. Results were tallied in advance of the conference.

**Conference Proceedings**

A full 1-day conference was held at the National Center for the Study of Preparedness and Catastrophic Response (PACER).^17^ Overview presentations were conducted by selected experts summarizing the current state of emergency department and hospital crowding, existing and novel surveillance mechanisms, and potential interventions to mitigate crowding as published in the peer-reviewed literature. The conference leaders then presented the results of the pre-conference survey and distributed these results to the panelists in tabular form. After these initial presentations, the panelists completed two exercises for which they were assigned to one of two equally sized groups, based upon panelists’ relevant expertise. One group evaluated ED-based interventions, and one evaluated hospital-based interventions. Each group was co-moderated by two subject matter experts (AD, MM, JP, JB, GK) with previous training through numerous instructional meetings and two mock moderation sessions to ensure uniform and unbiased facilitation of the proceedings.

During Exercise One, panelists were asked to prioritize potential interventions to mitigate surge in response to an infectious respiratory disease outbreak for each of four defined scenarios. Interventions were separated into two groups; those specifically for patients presenting with influenza-like illness (ILI) and those for patients without ILI. Infection control measures were included among the proposed interventions to focus efforts toward reducing disease spread and potentially reducing future patient volume. Using the nominal group technique, each of the two break-out groups (i.e. ED and hospital) was asked to discuss each associated intervention, (including the pre-panel “write-in” interventions), and suggest and similarly discuss any additional interventions not yet considered. Panel members were then asked to anonymously and independently categorize each intervention as low, medium, or high priority. Panelists were asked to consider both the ease of implementation and importance for a final overall priority consideration specific for each of the four respiratory infection outbreak scenarios.

In Exercise Two, panelists were asked to identify key data sources to trigger the implementation of a specific intervention in a given respiratory virus illness scenario. Considering the worst case scenario (high volume and high severity) and the recent 2009 H1N1 scenario (high volume, low severity), each group was given one intervention, which was highly rated in Exercise One, for each of the four scenarios. Additionally, each group was given a list of potential data sources based on those used in the literature, current medical practice, and current surveillance systems, but was also encouraged to consider and suggest alternate data sources that may serve as a “trigger” for implementation of the selected intervention. Examples of such data sources included ED wait times, hospital volume, laboratory data, syndromic and laboratory-based surveillance systems, and novel data sources that might be used to indicate increasing respiratory disease (e.g. Twitter). Panelists were asked to individually select the top three data sources that could be useful to ED or hospital leaders to “trigger” the decision to implement the top-rated interventions to mitigate ED or hospital crowding due to a respiratory infection outbreak. Similarly, using the nominal group technique, panelists proposed other potential data sources. All potential data sources were discussed and each panel member anonymously rated them on a 1-5 Likert scale with 1 being “not at all important” as a trigger and 5 as “very important” for a trigger for the selected interventions.

**Data Analysis**

To evaluate data from Exercise One, final categorization data were assigned a numerical score where Low Priority = 1, Medium Priority =3, and High Priority =5. These numerical scores were averaged by scenario to obtain a numerical final score for each intervention in each scenario. These final means were then categorized as either, Low Priority (1.0-2.0), Medium Priority (2.1-4.0), or High Priority (4.1-5.0). Additionally, numerical scores were averaged across all scenarios to obtain a total prioritization score for each intervention across all scenarios. Finally, scores for each intervention within a scenario were averaged to obtain a scenario prioritization score. For Exercise Two, the individual resultant ratings for each potential data source were averaged to obtain a mean “importance” score for each potential data source.

## Results

Among all of the potential interventions to mitigate ED crowding, the following interventions had the highest priority across all four outbreak scenarios: standardizing ED discharge and admission criteria for patients with respiratory symptoms (4.0), informing patients of the most appropriate setting for care either through a website (4.0) or telephone call center (3.9), and instituting infection control measures including hand sanitizers and masks (4.1), cohorting patients with ILI (4.0), or ED signage (3.9). Tables 1 and 2 demonstrate the final ED scenario-based prioritization for each potential intervention for both ILI patients (Table 1), and non-ILI patients (Table 2).

When considering a low volume/low severity scenario such as Scenario 1/Seasonal Influenza, no individual ED intervention ranked above medium priority which included standardization ED discharge and admission criteria for patients with respiratory symptoms (3.1), setting up a website to advise patients on the most appropriate setting for care (3.1), using hand sanitizer and masks (3.1), and ED signage for influenza (3.1). In the scenario with the highest volume and severity, Scenario 4/1918 H1N1, many interventions received high priority even if they had received a lower priority in all previous scenarios, such as; Opening an annex areas for patient care (4.7), coordination of influenza surge plans with the public health department (4.6), and protocols for non-medical volunteers (4.6).

Panelist ratings for hospital interventions identified the following as highest priority across all four outbreak scenarios: mandatory vaccinations (4.9), coordinating a public health message (4.1), creating a treatment algorithm for antiviral use (3.8), modifying visitation policies (3.7), screening employees (3.6), setting up alternate care systems (3.6), stockpiling isolation equipment (3.5), and setting up an inpatient quarantine area (3.5). Individual scenario-based ratings for the hospital interventions are shown in Tables 3 and 4.

The only hospital intervention to receive high priority in the low volume/low severity scenario, Scenario 1/Seasonal Influenza, was mandatory vaccinations (4.6). The remaining interventions received moderate or low prioritization. For the worst scenario with high volume/high severity, Scenario 4/1918 H1N1, all hospital interventions received high priority with the following interventions receiving the highest score of 5.0; modifying visitation policies, stockpiling isolation equipment for inpatient staff, creating a triage protocol for ventilator use, rescheduling or canceling elective procedures, and “double-bunking” inpatient rooms.

Overall, most individual ED-based interventions had higher priority ratings for scenarios which generated high patient volumes, such as Scenario 2/2009 H1N1 (3.6) and Scenario 4/1918 H1N1 (4.0), versus those with lower patient volumes, such as Scenario 1/Seasonal Influenza (2.0) and Scenario 3/SARS (3.0). Conversely, hospital-based interventions had higher priority ratings for scenarios with a high severity such as Scenario 3/SARS (3.8) and Scenario 4/1918 H1N1 (4.8) versus Scenario 1/seasonal influenza (1.8) and Scenario 2/2009 H1N1 (3.1).

The highest ranked interventions noted above were chosen for subsequent prioritization of potential data elements to employ as triggers (Table 5). ED interventions selected were: (1) establishing an ED annex (1918 H1N1 Scenario), and (2) constructing a website to advise patients on the most appropriate setting for care (2009 H1N1 Scenario). The hospital interventions selected were: (1) developing and implementing a ventilator triage protocol (1918 H1N1 Scenario), and (2) establishing alternate care sites (2009 H1N1 Scenario). The main data sources identified to serve as triggers came largely from medical center level crowding data as can be seen in Table 5. Top rated data sources consisted mainly of patient volume metrics (hospital volume, ED volume, boarding time in the ED, percent of patients with ILI, hospital capacity), patient acuity metrics (acuity ratio, ED admission rate, number of low acuity ILI patients), as well as measures of resource availability (ventilator supply, and staff availability).

## Discussion

Many EDs and hospitals are currently operating at or over capacity much of the time, where even small increases in patient volumes, such as those seen during seasonal influenza, worsen ED crowding and further prolong patient waiting times, which has been shown to be associated with untoward patient outcomes.^1^^,^ 6 ^,^ 7 ^,^ 8 ^,^ 9 ^,^ 10 ^,^ 18 ^,^ 19 ^,^ 20 A large-scale infectious respiratory disease outbreak requires advanced medical center planning and response in order to maintain good ED and hospital flow, and avoid any associated adverse impacts on patient care or outcomes. Although some interventions to manage ED and hospital crowding can be carried out at the institutional level, others, as noted by our expert panelists, require coordination with the local public health department, neighboring medical centers, or state and federal government to ensure alignment with the existing level of regulatory standards (e.g. suspension of the emergency medicine treatment and labor act [EMTALA]). Communication and coordination between all of these stakeholders increases the breadth of potential response options, and the overall effectiveness. Beyond the individual responses, these same organizations, with appropriate and timely sharing of data, combined with current surveillance systems, can inform the timing of when to implement the appropriate response. This expert panel sought to combine knowledge and experience of leaders in each of these fields to provide initial broad guidance and recommendations for prioritization and implementation of responses to mitigate crowding in the event of an infectious respiratory disease outbreak.

For ED settings, panelists gave overall higher priority to interventions in outbreak scenarios with high volume of patient visits, indicating the greatest concern for addressing outbreaks where ED crowding caused by sudden increased patient volume might worsen regardless of disease severity. To address a high patient volume outbreak, panelists prioritized several interventions, such as creating a website or telephone call center, to advise patients when to seek medical care and what the most appropriate setting for that care would be. Other highly ranked ED interventions were simple precautionary measures to protect non-ILI patients from being infected, such as hand sanitizers, masks, and cohorting infected patients, which are likely to have a delayed impact on ED crowding. Placing these highly rated interventions in the context of the often used ED crowding model of input, throughput and output, the majority of highly rated interventions focused on ED input, or reducing the number of patients presenting to the emergency department.^21^ Conversely, the majority of interventions presented in the peer-reviewed literature focus on interventions to impact ED throughput, or the speed at which ED patients are managed.^22^ Most of the literature evaluating the impact of interventions evaluates the combined impact of multiple interventions, making it difficult to isolate the effect of a single intervention. To our knowledge, only one paper isolates an ED input intervention showing that an ED vaccination campaign reduces ED visits for respiratory illness by 34%.^23^ Despite this evidence, this particular input intervention (ED vaccination) was rated lower by this expert panel, suggesting that cost and ease of implementation were important factors considered by the panel.

When considering hospital crowding, the panelists overall prioritized measures aimed at outbreaks with high severity. Unlike ED volume issues which are more related to overall patient volume, severity of illness, even with low volume of patients, stressed demand for inpatient resources such as hospital beds, intensive care unit (ICU) space and ventilators. Here, the highly rated interventions also focused on measures to protect staff and non-ILI patients from disease transmission, including setting up an inpatient quarantine area and stockpiling isolation equipment. This is consistent with previous task force recommendations to maximize ICU resources (both beds and staffing) as well as maintain strict infectious control policies.^24^ Additionally, a treatment algorithm for antiviral use rated highly, as antivirals would likely be in short supply and would require appropriate, scenario-driven allocation management.

For the low volume, low severity outbreak scenario (i.e. seasonal influenza), respondents gave higher prioritization to interventions that would be easier to implement (e.g. hand hygiene and masks), both for the ED and hospital. However, for either increased volume or severity outbreaks, respondents shifted prioritizations to those interventions requiring more intensive resources. For example, for the high-volume, high severity outbreak scenario (1918 pandemic), establishing an ED annex, stockpiling equipment, and adding staff were rated as highest priority.

This study evaluates the priority of interventions across a variety of potential infectious respiratory disease outbreak scenarios encompassing a range of patient volumes and disease severities. Although the exercise was designed to assess multiple epidemiological scenarios, it is often the case that the exact parameters of the outbreak (i.e. disease severity and patient volume) are not completely known at the outset of the outbreak. Thus, information gleaned from this exercise may be most helpful for understanding overall priority of interventions rather than their priority in a specific artificial scenario. However, given that there is often some advanced indication of the nature of an impending outbreak, and outbreak characteristics such as volume and severity affect prioritization of resulting interventions, detailed prioritization based on the volume and severity of an outbreak may prove beneficial.

Similar to other group consensus methods, results can vary based on the composition of the group. To mitigate this potential selection bias, we selected individuals across a wide range of expertise who are integral to the process of disease surveillance and implementation of clinical interventions. These selected individuals represented those in decision making positions from numerous geographic regions and organizations, academic institutions and levels of government. However, the results represent panelist’s opinions, and not the results of a formal literature meta-analysis or quantitative data analysis of U.S. hospitals; the inherent difficulty of which was described earlier in this article. Although further breakdown of the rankings according to the panelists self-identified expert group category would be an interesting analysis, this was not possible as several panelists identified themselves as belonging to more than one expert group, preventing clear categorization.

Information generated from these exercises may serve to guide ED and hospital administrators to focus on the highest-priority interventions for a particular scenario, based on anticipated patient volumes or disease severity. However, there is a need for more formal methods of assessing intervention quality and efficacy. Although conducting randomized controlled trials may not be possible for public health interventions, standardizing reporting and assessment of interventions could provide a framework for describing and comparing interventions which could be reported in the literature.

Although many of the highly rated interventions require, or would benefit from, coordination with departments of public health, local medical centers, and local and federal governments, the decision of whether and when to implement many of these interventions currently appears to occur at the medical center level. Further, virtually all of the most highly rated data sources to trigger the decision of when to implement these interventions are collected directly by individual hospitals or EDs. These data sources, such as ED and hospital volume and admission rates, and ED waiting times, are institutional data, monitored closely at the institutional level, but frequently have lags before reporting and analysis at the public health level. Despite emphasis on infectious respiratory disease surveillance, surveillance tools did not rank in the top three triggers for any of the four included scenarios. These recommendations highlight the importance of advancing capabilities of surveillance systems to collect and coalesce institutional data sources in a timely manner, to provide both public health departments and medical institutions with better situational awareness which could improve the timeliness and effectiveness of planned responses.

## Conclusion

Expert panelists placed highest priority on ED interventions to mitigate surge in the setting of an infectious respiratory disease outbreak scenario with high patient volume, indicating the greatest concern for addressing outbreaks where ED crowding caused by sudden increased patient volume might worsen regardless of disease severity. Disease severity, in and of itself, was felt to be a lesser issue in the ED setting. Conversely, panelists concluded that interventions addressing disease severity were the most important for inpatient management, operations and resource distribution, with less emphasis decreasing total numbers affected in the population. Data useful for setting triggers to implement high priority measures were predominantly those available at the local institutional level. Strikingly, public health surveillance data were not considered as one of the top triggers to operationalize any of the priority measures, either for the ED or the inpatient services. We believe this study represents the first step in delineating high impact interventions according to hospital service (ED verses inpatient) and infectious disease outbreak characteristics.

**Scenario based prioritization for interventions to mitigate emergency department (ED) crowding in the event of an infectious respiratory virus outbreak designed to impact patients presenting with influenza-like illness (ILI):**
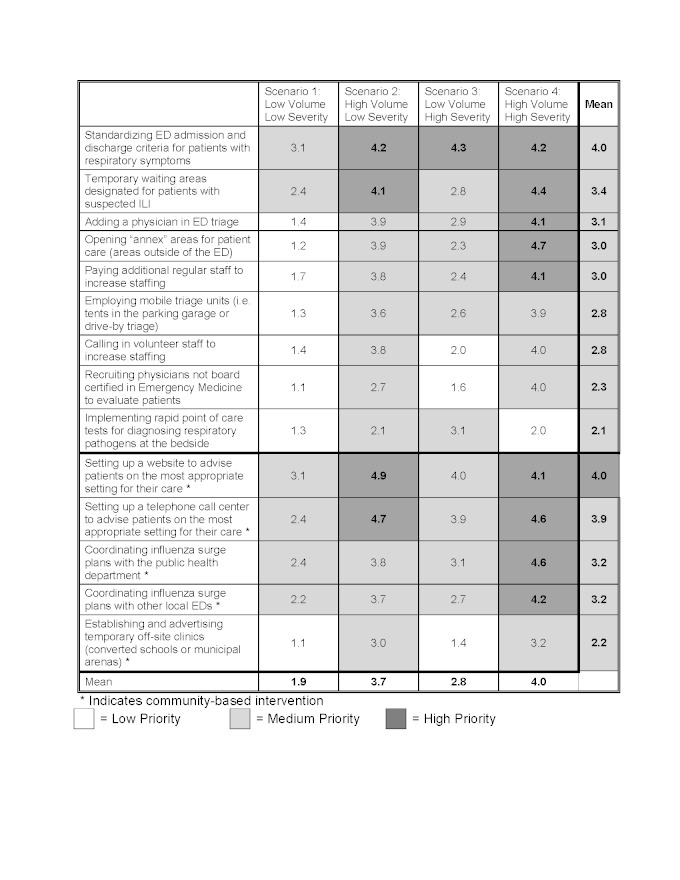


**Scenario based prioritization for interventions to mitigate emergency department (ED) crowding in the event of an infectious respiratory virus outbreak designed to impact patients without influenza-like illness (ILI):**
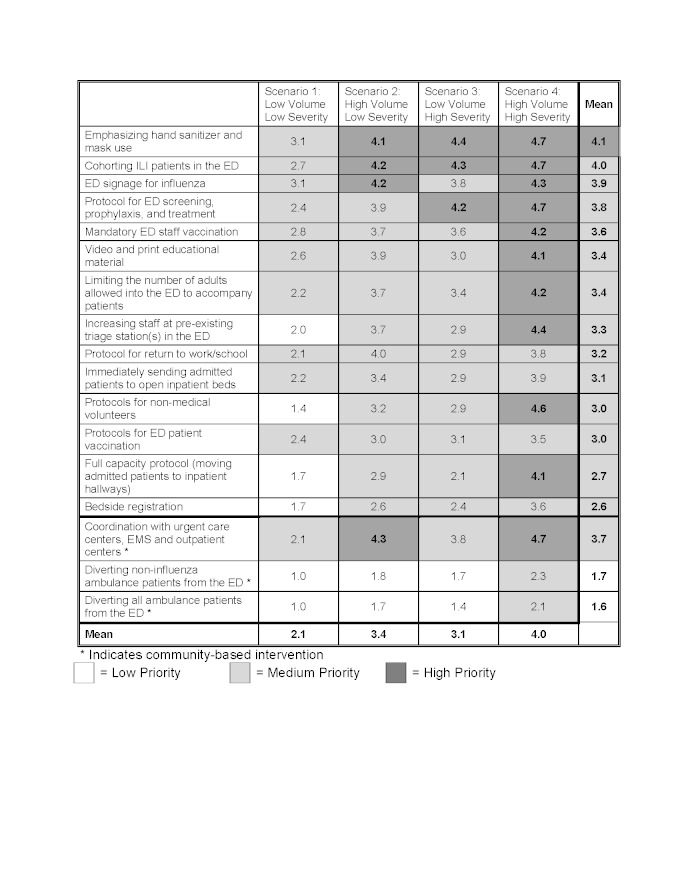


**Scenario based prioritization for interventions to mitigate hospital crowding in the event of an infectious respiratory virus outbreak designed to impact patients presenting with influenza-like illness (ILI):**
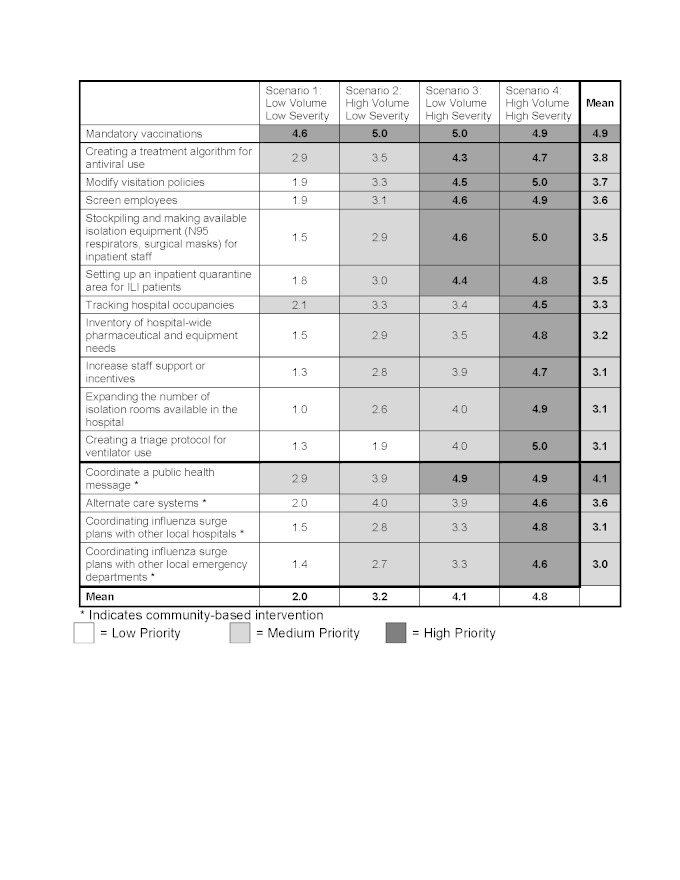


**Scenario based prioritization for interventions to mitigate hospital crowding in the event of an infectious respiratory virus outbreak designed to impact patients presenting without influenza-like illness (ILI):**
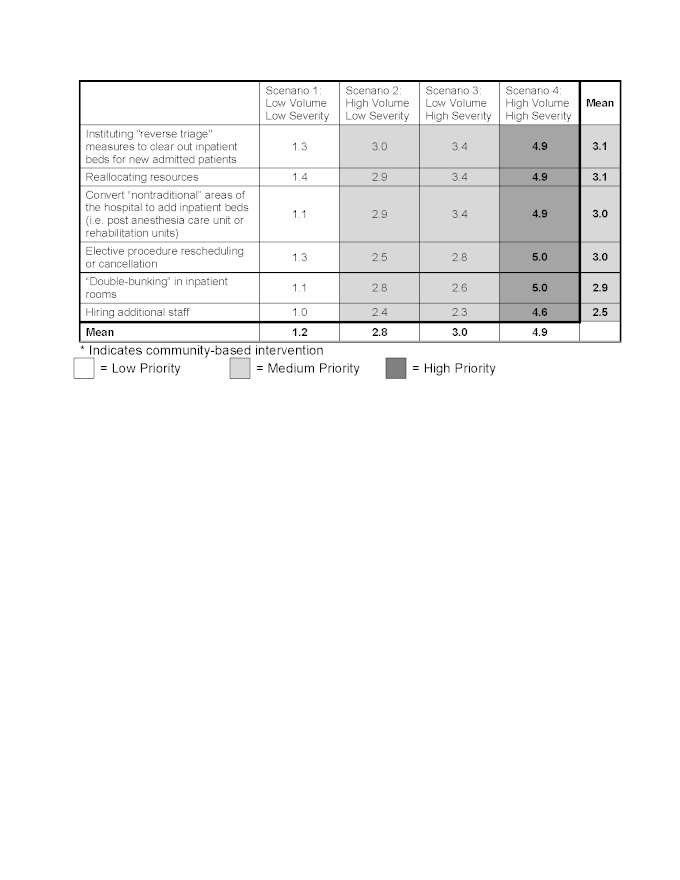


**Linking Data Sources to Intervention Implementation:**
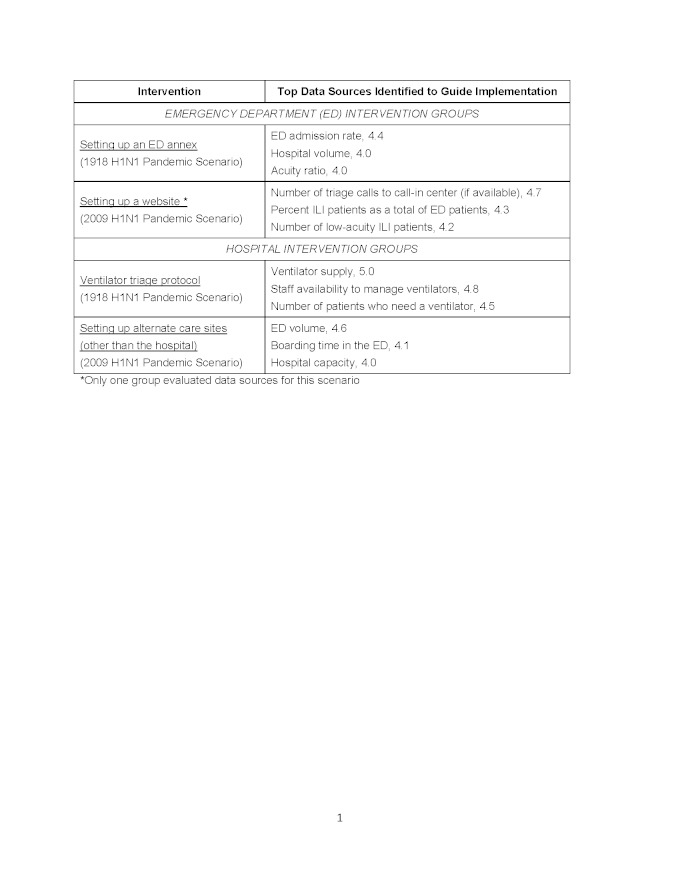


## Competing Interests

The author have declared that no competing interests exist
